# Novel therapeutic for multiple sclerosis protects white matter function in EAE mouse model

**DOI:** 10.3389/fmmed.2023.1237078

**Published:** 2023-08-28

**Authors:** Sarah Zerimech, Hung Nguyen, Arthur A. Vandenbark, Halina Offner, Selva Baltan

**Affiliations:** ^1^ Anesthesiology and Perioperative Medicine (APOM), Oregon Health and Science University, Portland, OR, United States; ^2^ Neuroimmunology Research, VA Portland Healthcare System, Portland, OR, United States; ^3^ Department of Neurology, Oregon Health and Science University, Portland, OR, United States; ^4^ Department of Molecular Microbiology and Immunology, Oregon Health and Science University, Portland, OR, United States

**Keywords:** microglia, astrocyte, axon function, CAP, myelin, DRHQ

## Abstract

Multiple sclerosis (MS) is a chronic demyelinating disease with prominent axon dysfunction. Our previous studies in an MS mouse model, experimental autoimmune encephalomyelitis (EAE), demonstrated that major histocompatibility complex Class II constructs can reverse clinical signs of EAE. These constructs block binding and downstream signaling of macrophage migration inhibitory factors (MIF-1/2) through CD74, thereby inhibiting phosphorylation of extracellular signal-regulated kinase (ERK) activation and tissue inflammation and promoting remyelination. To directly assess the effects of a novel third generation construct, DRhQ, on axon integrity in EAE, we compared axon conduction properties using electrophysiology on corpus callosum slices and optic nerves. By using two distinct white matter (WM) tracts, we aimed to assess the impact of the EAE and the benefit of DRhQ on myelinated and unmyelinated axons as well as to test the clinical value of DRhQ on demyelinating lesions in CC and optic myelitis. Our study found that EAE altered axon excitability, delayed axon conduction and slowed spatiotemporal summation correlated with diffuse astrocyte and microglia activation. Because MS predisposes patients to stroke, we also investigated and showed that vulnerability to WM ischemia is increased in the EAE MS mouse model. Treatment with DRhQ after the onset of EAE drastically inhibited microglial and astrocyte activation, improved functional integrity of the myelinated axons and enhanced recovery after ischemia. These results demonstrate that DRhQ administered after the onset of EAE promotes WM integrity and function, and reduces subsequent vulnerability to ischemic injury, suggesting important therapeutic potential for treatment of progressive MS.

## Introduction

Multiple sclerosis (MS) is a demyelinating and inflammatory disease of the central nervous system (CNS), which affects over 2 million patients in the world globally (Wallin et al., 2019). Based on the course of the disease, four major types of MS have been described: 1) Relapsing-Remitting MS (RRMS) is the most common and affects 85% of the patients with symptoms of demyelination, followed by periods of remission. 2) Among all MS patients, 30% will develop a Secondary Progressive MS (SPMS) with demyelination symptoms without periods of remission. 3) Patients can develop a progressive type of MS, called Primary Progressive MS (PPMS) that is more resistant to the usual treatment for MS. 4) A fourth and rarer form of MS is Progressive-Relapsing MS (PRMS), described as a progressive form with intermittent deteriorating symptoms without remission (Steinman, 2001; Goldenberg, 2012). Breakdown of the blood–brain barrier (BBB), multifocal inflammation, demyelination in gray and white matter (WM), oligodendrocyte loss, reactive gliosis, and axonal degeneration occurs in MS lesions (Prineas et al., 2001; Dutta and Trapp, 2007; Trapp and Nave, 2008; Dutta and Trapp, 2014). Both genetic and environmental factors are involved in MS disease pathogenesis and progression. The main genetic effect has been attributed to the major histocompatibility complex (MHC) region on chromosome 6, which encodes class I and class II proteins that present “self” myelin peptides to activate encephalitogenic CD4^+^ and CD8^+^ T-cells. Non-polymorphic MHC proteins such as class II invariant chain (CD74, II) and HLA-DRα chain, contribute to the genetic effect. CD74 chaperones peptide-loaded MHC Class II molecules from intracellular compartments to the surface antigen-presenting cells (APC). CD74 also functions as the receptor for the macrophage migration inhibitory factor (MIF-1) and its homologue D-dopachrome tautomerase (D-DT = MIF-2) when it is expressed on the cell surface or secreted in a soluble form. MIF is a pleiotropic innate cytokine and is a key mediator of various inflammatory diseases including MS. In patients with MS increased MIF levels are detected in the blood and CSF during relapse, and in the active rim of MS WM lesions (Meza-Romero et al., 2014; Benedek et al., 2015; Benedek et al., 2017; Vandenbark et al., 2021).

Experimental autoimmune encephalomyelitis is a MS mouse model commonly used to develop and validate disease-modulating therapies. EAE pathology relies on external sensitization of the animal to myelin antigens, leading to the development of inflammation and demyelination. EAE can be induced in two main genetic backgrounds in mice; SJL and C57BL6, by using antigens such as myelin oligodendrocyte glycoprotein (MOG), proteolipid protein (PLP), myelin basic protein (MBP), and others mixed in complete Freund’s adjuvant (CFA). In C57BL6 mice, the use of MOG in CFA leads to the development of a chronic progressive pathology; while in the SJL mouse model, a relapsing-remitting form of EAE can be induced with PLP (Constantinescu et al., 2011; Glatigny and Bettelli, 2018).

DRα1 (L50Q)-MOG-35-55 (DRhQ) is a partial Class II MHC construct comprised of the extracellular MHC class II L50Q substituted DRα1 subunit linked to the human MOG-35-55 peptide (myelin antigen). It is a third generation of recombinant T-cell receptor ligand (RTL) constructs, based on an RTL1000 construct designed with DRα1 and DRβ1 subunits linked covalently to MOG-35-55. Studies showed that DRα1 but not DRβ1 binds to CD74, leading to macrophage inactivation, and preventing MIF-1/D-DT ligands from binding to CD74, thereby inhibiting downstream MAPK pathway activation (Vandenbark et al., 2019). Both the L50Q substitution in the DRα1 domain and the covalent extension of the construct with MOG-35-55 peptide leads to a higher affinity binding to CD74 and stronger competitive inhibition of MIF/DDT binding (Yang et al., 2017; Meza-Romero et al., 2019; Vandenbark et al., 2019; Vandenbark et al., 2021; Vandenbark et al., 2022).

Assessment of clinical EAE behavioral test scores showed that DRhQ improves clinical motor sign scores in a murine model of MS (EAE) (Meza-Romero et al., 2014; Benedek et al., 2015; Vandenbark et al., 2021). DRhQ also inhibits binding of MIF to CD74, a key inflammatory factor involved in various immune-mediated diseases (Meza-Romero et al., 2014; Benedek et al., 2015). An enhanced cell surface expression of CD74 has been demonstrated both in EAE-induced animal models and MS patients (Benedek et al., 2015). DRhQ reduces the number of infiltrating cells in CNS and reduces their activation. Moreover, DRhQ reduces the expression of pro inflammatory genes and increases the expression of genes involved in neuronal survival and regeneration (Meza-Romero et al., 2014; Benedek et al., 2015; Benedek et al., 2017; Vandenbark et al., 2021; Vandenbark et al., 2022).

In our present study, using *ex vivo* electrophysiological recording, we directly assessed the effect of the DRhQ construct on the axon function in two different WM tracts: the corpus callosum (CC) and optic nerves in a murine model of MS (EAE) with or without treatment. Optic nerve is a pure WM tract consisting of fully myelinated axons while CC neighbors’ gray matter and consists of a mixture of myelinated and unmyelinated axons (Seggie and Berry, 1972; Sturrock, 1980; Preston et al., 1983; Hildebrand et al., 1993; Nunez et al., 2000; Tekkok and Ransom, 2004). Both WM tracts are targets of MS attacks and represent hallmarks of the disease pathology such as optic neuritis (Chan, 2002; Arnold, 2005; Kale, 2016) and demyelinating lesions (Breij et al., 2008; Kutzelnigg and Lassmann, 2014). Evoked compound action potentials (CAPs) were recorded under control conditions to characterize the axon conduction properties. Then, using an oxygen glucose deprivation (OGD) protocol, the impact of EAE pathology on a subsequent ischemic injury and whether DRhQ attenuates the subsequent WM injury were tested. We showed that EAE pathology alters axon conduction properties and that DRhQ restores it to control values. Furthermore, EAE renders WM axon function more vulnerable to a subsequent ischemic injury, while DRhQ treatment attenuates this susceptibility. This suggests that DRhQ administered after EAE onset promotes WM integrity and function, and reduces vulnerability to ischemic injury by attenuating neuroinflammatory responses.

## Materials and methods

### Animals

Experiments were carried out according to the Institutional Animal Care and Use Committees (IACUC) of Oregon Health & Science University and VA Portland Healthcare System and performed and reported in compliance with ARRIVE guidelines (Animal Research: Reporting *In Vivo* Experiments). C57BL/6 WT male mice between 8 and 12 weeks of age were purchased from the Jackson Laboratory. All mice were maintained on a 12-h light/dark cycle with food and water provided *ad libitum*. All efforts were made to minimize the number of animals used and their suffering.

### EAE induction

**FIGURE 1:**
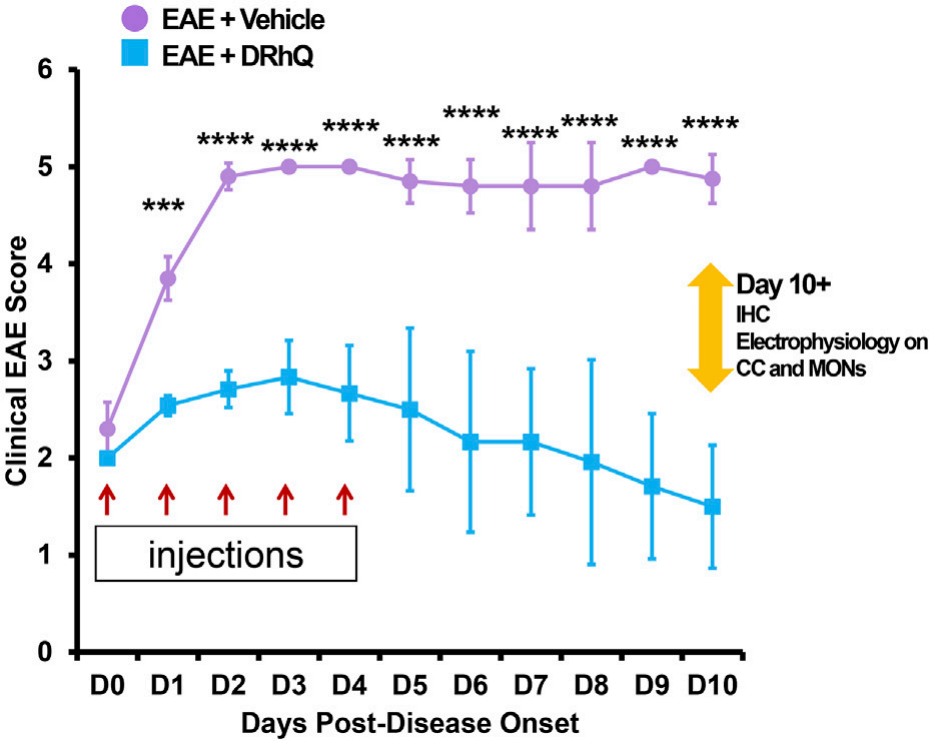
Treatment with DRhQ decreases severity of acute EAE resulting in significantly improved clinical scores. The mice were scored for clinical signs of EAE graded on a six-point scale of combined hindlimb and forelimb paralysis scores. After immunization with an emulsion of 200 µg mouse MOG-35-55 peptide and 400 µg complete Freund’s adjuvant + Ptx on Days 0 (75 µg) and 2 (200 µg), mice were treated with either vehicle or DRhQ (100 µg in 0.1 ml) by subcutaneous injection daily for 5 days beginning at an EAE score of 2.0. In Figure 1, D0 (day zero) represents the beginning of the treatment. At D1, treatment with DRhQ diminished clinical EAE scores: EAE + vehicle, n = 5: 3.8 ± 0.1; EAE + DRhQ, n = 6: 2.5 ± 0.04, ***p = 0.0007. At D10 treated mice with DRhQ had significant lower EAE score: EAE + vehicle (n = 4): 4.8 ± 0.1, EAE + DRhQ (n = 6): 1.5 ± 0.2, ****p < 0.0001. Two-way ANOVA, Bonferroni’s correction. Data are presented as mean ± SEM.

Mice were immunized subcutaneously at four sites on the flanks to distribute a 0.2 ml emulsion of 200 µg mouse MOG-35-55 peptide and complete Freund’s adjuvant containing 400 µg of heat-killed *Mycobacterium tuberculosis* H37RA (Difco) (Vandenbark et al., 2003; Meza-Romero et al., 2014; Meza-Romero et al., 2019). Mice were also injected intraperitoneally with Pertussis toxin (Ptx) from List Biological Laboratories on days 0 and 2 post-immunization (75 and 200 ng per mouse, respectively); Ptx is used as an accessory adjuvant in EAE induction (Munoz et al., 1981; Noubade et al., 2008). DRhQ (100 µg in 0.1 ml) was injected subcutaneously daily for 5 days beginning at an EAE score of >2.0. The mice were scored for clinical signs of EAE graded on a six-point scale of combined hindlimb and forelimb paralysis scores as described in a previous study (Meza-Romero et al., 2014; Vandenbark et al., 2021; Vandenbark et al., 2022; Offner et al., 2023) (Figure 1).

### Slice preparation

Acute coronal brain slices were prepared as previously described (Tekkok and Goldberg, 2001; Tekkok and Ransom, 2004; Tekkok et al., 2005). After a deep CO2 asphyxiation, mice were sacrificed by decapitation. The brains were quickly dissected and placed in an ice-cold artificial cerebrospinal fluid (standard ACSF) composed of (mM): 126 NaCl, 3.5 KCl, 1.3 MgCl2, 2 CaCl2, 1.2 NaH2PO4, 25 NaHCO3, and 10% glucose, pH 7.4, with a mixture of 95% O2/5% CO2. The whole brain was placed on a vibrating blade microtome (Leica, VT1000S, IL). After dissecting the cerebellum, the brain is placed and glued (Tissue Adhesive 3M Vetbond) in front of an agarose cube (2% prepared in ASCF) with its inferior surface facing the agarose cube. This orientation stabilized the brain at approximately 20–22° to its vertical axis. Acute coronal slices of 300 µm were prepared. Only the slices (8-10 per brain) with clearly visualized anatomical structure of the CC were collected. Slices were kept in resting chamber for at least 2 h at room temperature in standard ACSF saturated with 95%O2/5%CO2. Slices were then placed on a piece of lens paper and transferred to a Haas-type chamber (Harvard Apparatus, Holliston, MA) and kept at an interface between a warm humidified gas (95% O2/5%CO2) and oxygenated ACSF at 33°C ± 1 °C with a flow rate of 3–3.5 ml/min. The superfusion and chamber temperatures were controlled by a Dual Channel Heater Controller (Model TC-344B, Harvard Apparatus, Warner Instruments, MA). Each slice was kept in the chamber for at least 30 min to stabilize before baseline responses were recorded.

### Optic nerve preparation

Following CO2 asphyxiation, mouse optic nerves (MONs) were gently collected from mice by an experimenter blinded to their identity. The MONs were transferred to a Haas-type chamber (Harvard Apparatus, Holliston, MA) and were allowed 15 min to stabilize while continuously being aerated by a humidified gas mixture of 95%O_2_/5%CO_2_. All recordings were performed at 37°C. The superfusion and chamber temperatures were controlled by a Dual Channel Heater Controller (Model TC-344B, Harvard Apparatus, Warner Instruments, MA).

### Compound action potential recordings across corpus callosum and optic nerve axons

Extracellular compound action potentials (CAP) across the corpus callosum were evoked every 30 s by supramaximal pulses of 1 mA (30 μs) duration using a specifically designed bipolar stimulation electrode (A365R stimulus isolator; WPI, Sarasota, FL). The evoked CAPs were recorded with borosilicate glass micropipettes filled with 2M NaCl, connected to an Axoclamp 900A amplifier and Digidata 1440B (Molecular Devices, LCC, Sunnyvale, CA). The signal was amplified 50 times (SR560; Stanford Research Systems, Sunnyvale, CA) and acquired at 10 kHz. The responses were digitized, stored, and quantified by measuring the area under the CAPs. Baseline CAP responses were recorded for 30 min. To mimic ischemic injury, oxygen glucose deprivation (OGD) was induced for 30 min by replacing ACSF with glucose-free ACSF (10 mM glucose replaced with 10 mM sucrose) saturated with 95%N2/5%CO2. Slices were superfused with glucose containing oxygenated ACSF for another 30 min after the end of OGD.

The CAPs across MONs were recorded by using suction electrodes backfilled with ACSF. MONs were stimulated via stimulus isolation unit (Isostim 520; WPI, Sarasota, FL) and the resultant CAPs were acquired by a recording electrode connected to an Axoclamp 2A amplifier. The signal was amplified 50 times, filtered at 30 kHz, and acquired at 20–30 kHz. Stimulus pulse (30 µs duration) strength was adjusted to evoke the maximum CAP possible at every 30 s interval, and then increased another 25% for supramaximal stimulation. The OGD was induced for 60 min as described above. The CAPs were recorded another 3–5 h after the end of OGD. The experimenter was blinded to the identity of the mice during the experiments.

### Immunohistochemistry

The experiments were performed in perfusion-fixed (4% paraformaldehyde in PBS) MONs or coronal brain slices in a mixture of 4% paraformaldehyde (#P6148, Millipore Sigma, St-Louis, MO) in PBS and 0.025% glutaraldehyde (#O2957, Fisher Scientific, Pittsburgh, PA). Cryoprotection was achieved in 30% sucrose for 16–18 h. Sections of 50 µm (brains) and 16 µm (MONs) thickness were blocked in 20% normal goat/donkey (50% by volume) serum (#G9663 & G6023, Millipore Sigma, St-Louis, MO) and 1% Triton X-100 (#X100, Millipore Sigma, St-Louis, MO) for 60 min at room temperature. Sections were then incubated in primary antibodies prepared in the same solution and kept overnight at 4°C (see Table 1 for antibodies and dilutions used). After several thorough washes in PBS, the tissue was incubated with secondary antibodies, prepared in 2% normal goat serum for 2 h at room temperature. The secondary antibodies used were donkey anti-rabbit Cy2, anti-mouse Cy2 and anti-rabbit Cy3 and anti-mouse Cy3 (Jackson ImmunoResearch Laboratories, Inc., West Grove, PA). Sections were double or triple labeled to visualize structures of interest.

**TABLE 1:** Antibodies and dilutions.

Name	Dilution	Vendor	Catalog
Iba-1	1:100	Wako	019-19741
GFAP	1:15	Immunostar	22522
NF200	1:100	ThermoFisher	13-1300
MBP	1:2000	Millipore-Sigma	AB5864
Olig-2	1:100	ThermoFisher	P21954

### Confocal microscopy and pixel intensity measurements

The expression of GFAP (Glial Fibrillary Acidic Protein) (+) astrocytes, Iba (+) microglia, NF160/200 (+) axons, MBP (+) myelin and Olig-2 (+) oligodendrocytes were imaged by using an Olympus FV3000 inverted confocal laser-scanning microscope (Center Valley, PA) with 5 OBIS laser diode lines (405nm, 445nm, 488nm, 514nm and 640 nm) (Santa Clara, CA). Sections were scanned for Cy2/488, and for Cy3/514. Two to three adjacent sections from each sample were imaged for five areas of interest (AOI). A total of 10–16 optical sections of 1 μm thickness at 1024 × 1024-pixel size were collected in the *z*-axis from a single microscopic field using a 40X (UPLSAPO 40XS, silicone immersion; numerical aperture, 1.25; Olympus, Center Valley, PA) objective lens under fixed gain, laser power, pinhole, and photomultiplier tube (PMT) settings. To compare and quantify immunohistochemical staining, all sections were processed concurrently. Images were acquired with Olympus FluoView imaging software in sequential mode using multiple channels simultaneously. Z-stacks were projected into a single plane image before analysis and assessment of pixel intensity or co-localization. Final images were produced by either original or artificial color.

### Data analysis

**FIGURE 2:**
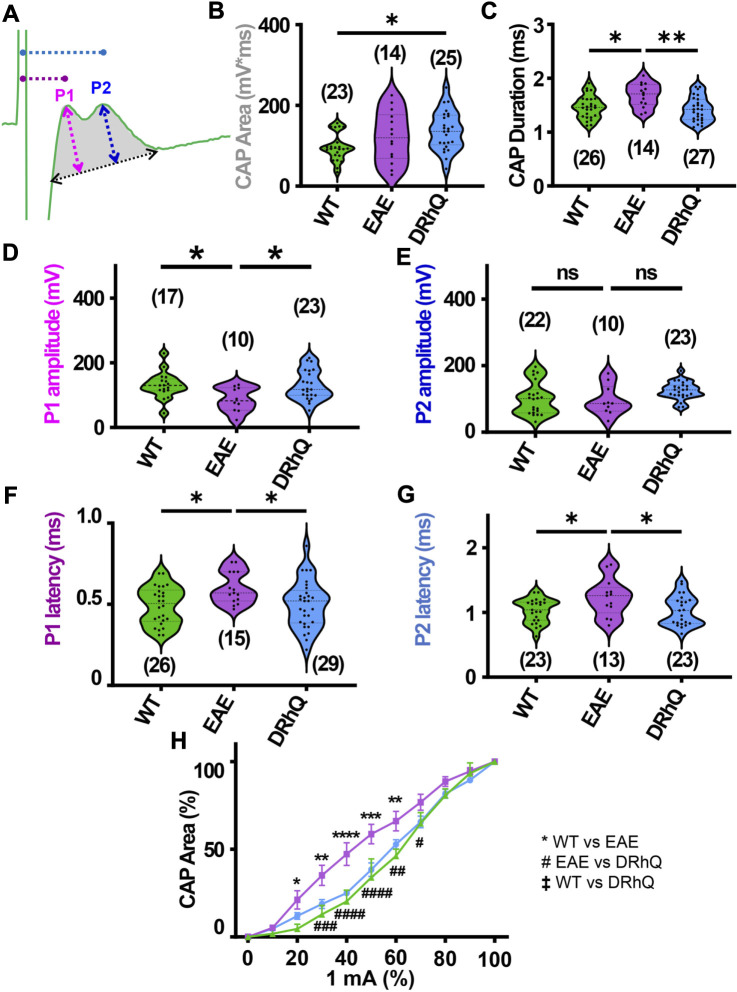
EAE alters axon conduction properties. DRhQ restores axon function. CAPs were recorded for 30 min under baseline conditions. Wild type (WT) is indicated in green, EAE in purple, and DRhQ in blue. **(A)** Baseline axon function (depicted in gray shaded area) is measured as the mean of the absolute CAP area during the last 10 min of baseline, action potential amplitude (mV) as a function of spatiotemporal summation measured as CAP duration (ms) (black dotted arrow). **(B)** Absolute CAP area represents the axon function. WT: 95.6 ± 7.03, EAE: 122.3 ± 16.8, DRhQ: 137.2 ± 9.7, *p = 0.0133, one-way ANOVA; WT versus DRhQ *p = 0.0108. **(C)** Spatiotemporal distribution of axons’ action potentials is measured as the duration of the signal in baseline condition. WT: 1.46 ± 0.04, EAE: 1.7 ± 0.06, DRhQ: 1.4 ± 0.04, one-way ANOVA; WT versus EAE, *p = 0.0106, EAE versus DRhQ **p = 0.0016. **(D, E)** Amplitude of the myelinated and unmyelinated axons is measured respectively as the amplitude of the first and second peak. **(D)** WT: 130.7 ± 9.8; EAE: 86.5 ± 11.8; DRhQ: 130.2 ± 9.5, *p = 0.0187. **(E)** WT: 101.0 ± 10.5; EAE: 95.8 ± 14.3; DRhQ: 124.9 ± 5.5, *p* = 0.0758. **(F, G)** Conduction velocity of myelinated and unmyelinated axons is measured as the latency between the stimulation artifact and the first or the second peak. **(F)** WT: 0.5 ± 0.02, EAE: 0.6 ± 0.02, DRhQ: 0.5 ± 0.02, one-way ANOVA; WT versus EAE *p = 0.0294, DRhQ versus EAE *p = 0.04. **(G)** WT: 1.0 ± 0.04, EAE: 1.2 ± 0.08, DRhQ: 1.0 ± 0.05, *p = 0.02, one-way ANOVA; WT versus EAE *p = 0.0385, DRhQ versus EAE *p = 0.0437. **(H)** Axon excitability is measured as the evoked CAP area in response to increasing intensity, from 0% to 100% of 1 mA. Significant differences between WT and EAE are represented with *, and differences between EAE and DRhQ are represented by #; F_treatment_ = 1.517, ****p < 0.0001, two-way ANOVA. Two-way ANOVA and one-way ANOVA with a Bonferroni’s post-hoc correction was used for the statistical analysis and data is presented as mean ± SEM.

**FIGURE 3:**
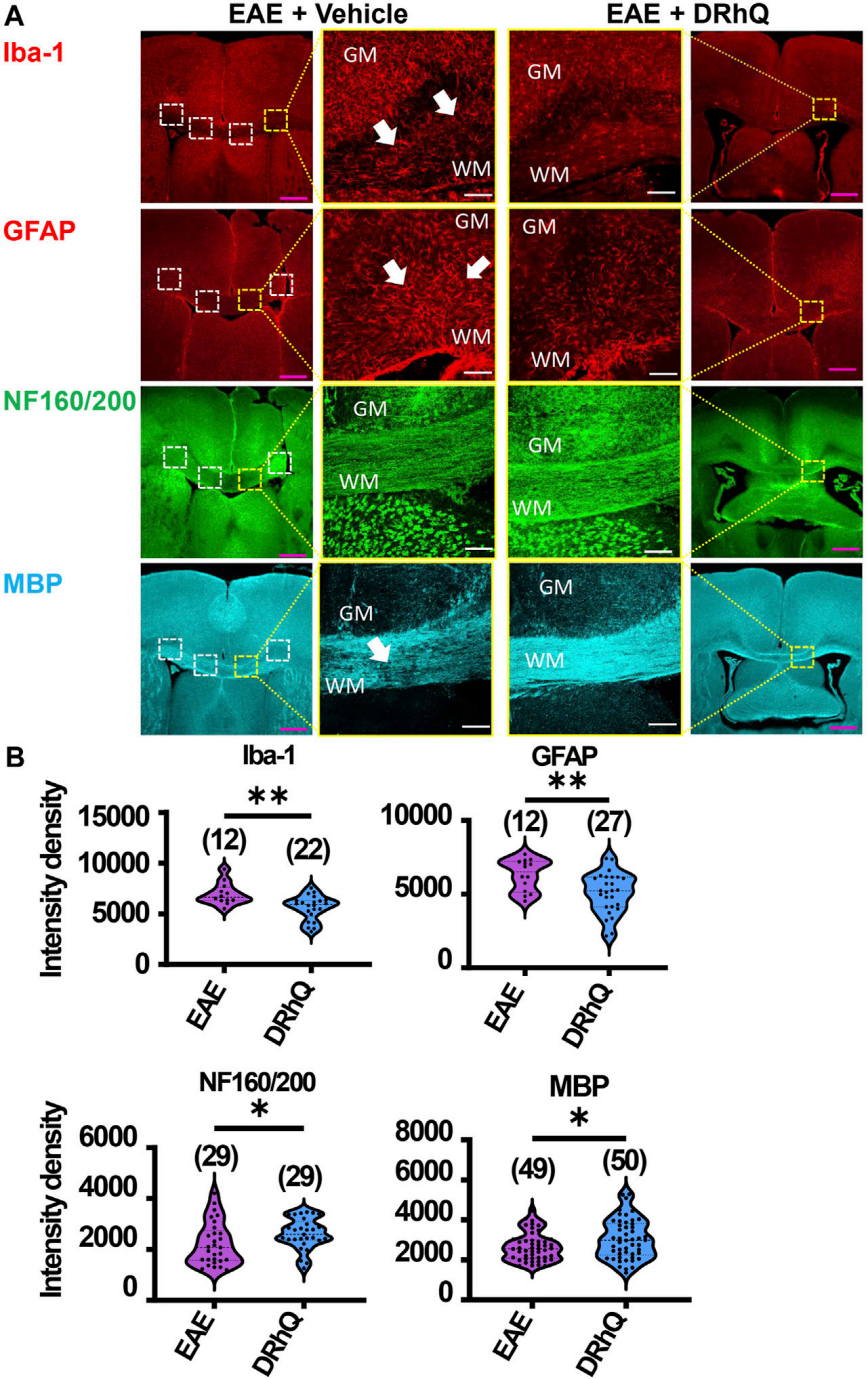
EAE activates microglia and astrocytes, causes axon and myelin loss. DRhQ alleviates EAE-mediated structural alteration. **(A)** EAE activates microglia and astrocytes in white matter seen as the high expression of Iba-1 (+) and GFAP (+) cells, respectively. Treatment substantially reduces activated microglia and astrocytes seen in white matter. EAE has modest effects on corpus callosum axons indicated by neurofilament staining and treatment has modest but significant protective effects. EAE damages myelin seen in the loss of MBP staining. Treatment drastically preserves myelin against EAE induced injury. 4x images with 500 µm pink scale bar; 20x images with 100 µm white scale bar. ROI used for quantification and analysis in **(B)** are represented as white dotted squares. **(B)** Quantitative analysis of the staining density for Iba-1, GFAP, NF160/200 and MBP labeling in EAE + vehicle and EAE + DRhQ CC slices. DRhQ treatment significantly decreased intensity density for Iba-1, GFAP while increased intensity density for NF160/200 and MBP labelling. Two-tailed Student’s t-test was used for the statistical analysis. EAE is indicated in purple and DRhQ in blue.

**FIGURE 4:**
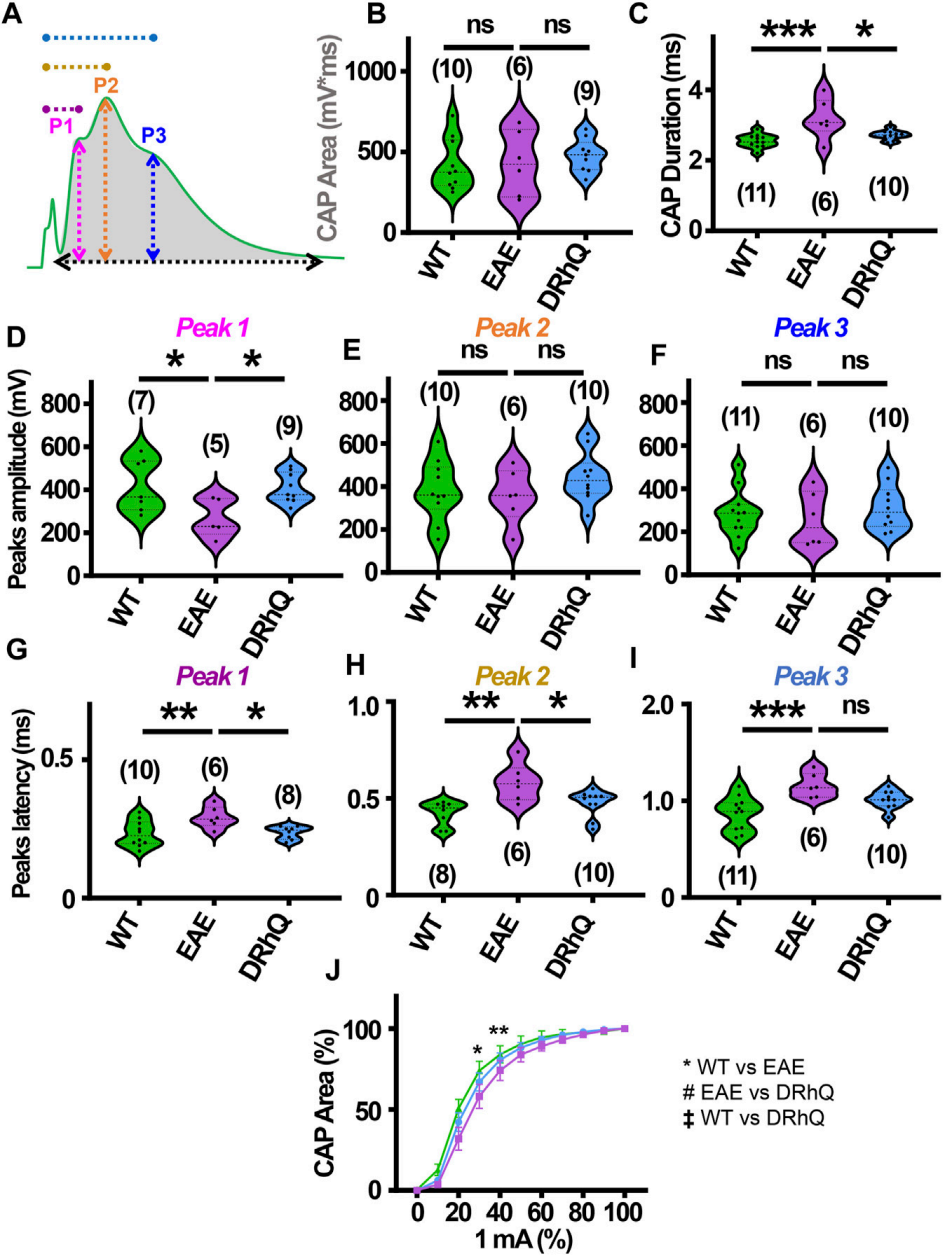
Wild type (WT) is indicated in green, EAE in purple, and DRhQ in blue. **(A)** Baseline axon function is measured as the mean of the absolute CAP area during the last 10 minutes of baseline. CAPs with three peaks (P); P1, depicted with a pink line, P2 depicted by an orange line, and P3 depicted by a blue line. (**B**) Absolute CAP area represents the axon function. WT: 418.9 ± 50.1, EAE: 430.6 ± 81.2, and DRhQ: 478.2 ± 33.9, ns *p* = 0.6829. **(C)** Spatiotemporal distribution of axons’ action potentials is measured as the duration of the signal in baseline condition. WT: 2.54 ± 0.06, EAE: 3.18 ± 0.2 DRhQ: 2.71 ± 0.04, ** *p* = 0.0011. **(D, E, F)** Amplitude of the myelinated, intermediate, and unmyelinated axons is measured respectively as the amplitude of the first, second and third peak. **(C)** P1: WT: 419.5 ± 121.9, EAE: 266.9 ± 89.1; **(D)** P2: WT: 381.1 ± 43.7, EAE: 356.1 ± 51.5; **(E)** P3: WT: 284.8 ± 33.2, EAE: 256.0 ± 51.4. **(G, H, I)** Conduction velocity of myelinated, intermediate, and unmyelinated axons is measured as the latency between the stimulation artifact and the first, the second or third peak. **(G)** Time to P1, WT: 0.2 ± 0.01, EAE: 0.3 ± 0.01, DRhQ: 0.2 ± 0.01, * *p* = 0.0161; **(H)** Time to P2, WT: 0.4 ± 0.02, EAE: 0.6 ± 0.04, DRhQ: 0.5 ± 0.02, ** *p* = 0.0026; **(I)** Time to P3, WT: 0.9 ± 0.05, EAE: 1.2 ± 0.05, DRhQ: 1.0 ± 0.03, ** *p* = 0.0012. **(J)** Axon excitability is measured as the evoked CAP area in response to increasing intensity, from 0 to 100% of 1 mA. Significant differences between WT and EAE are represented with *, F_treatment_ = 0.2940, ** *p* = 0.0014. Two-way ANOVA and one-way ANOVA with a Bonferroni’s post-hoc correction was used for the statistical analysis and data is presented as mean ± SEM.

**FIGURE 5:**
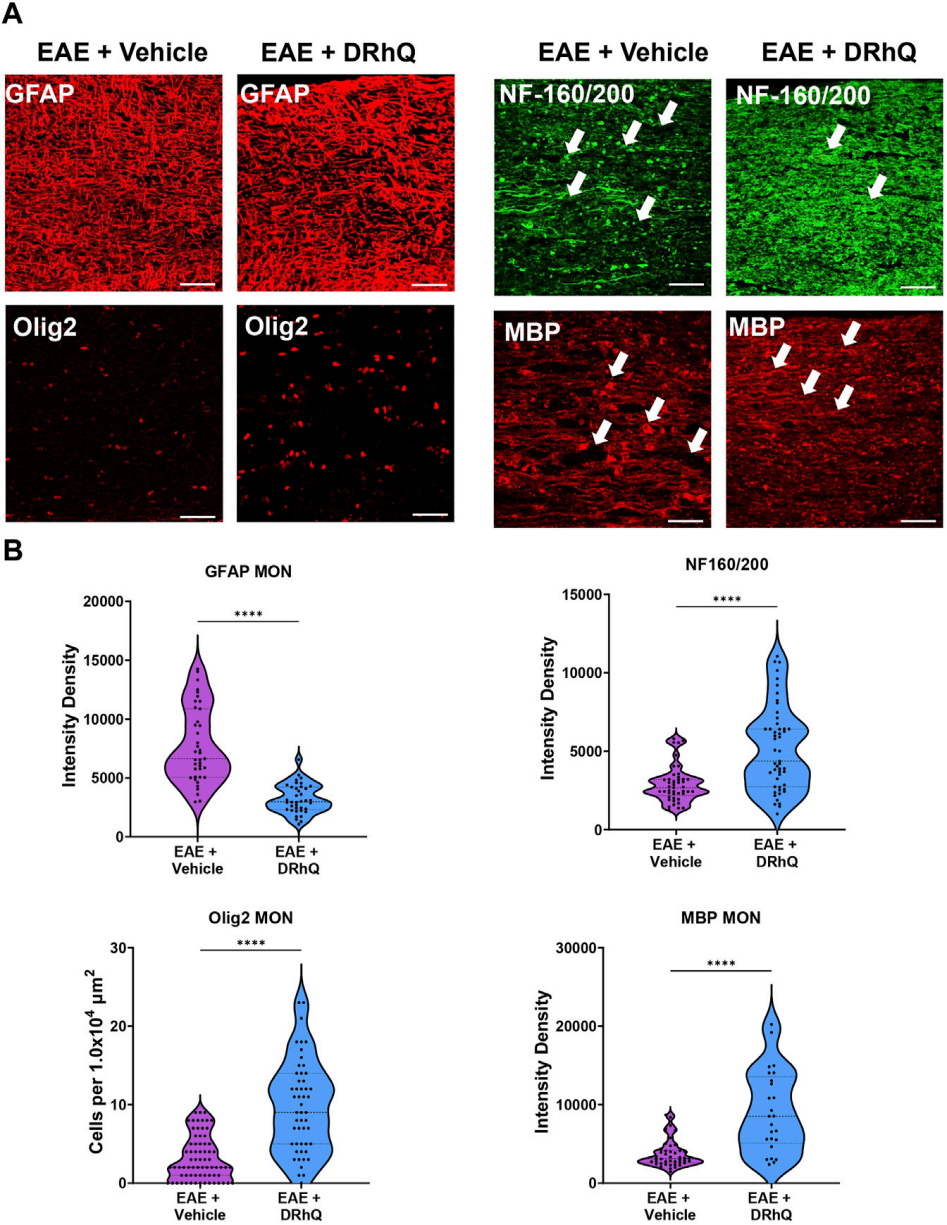
EAE damages axons in optic nerves. DRhQ substantially reduces EAE induced injury in white matter. **(A)** EAE drastically damages axons in optic nerve indicated by NF160/200 (+) neurofilament staining. EAE activates astrocytes in optic nerve seen as the bright expression of GFAP (+) cells with astrogliosis morphology. EAE causes abundant damage to myelin and oligodendrocytes seen in the loss of MBP staining and ghostly Olig-2 staining. Treatment drastically reduced EAE induced injury in optic nerve. 40x images with 50 µm scale bar. **(B)** Quantitative analysis of the staining density for GFAP, NF160/200, APC and MBP labeling in EAE + vehicle and EAE + DRhQ in optic nerves. DRhQ treatment significantly decreased intensity density for GFAP while increased intensity density for NF160/200 MBP labelling, and increased Olig-2 (+) oligodendrocyte cell count. Two-tailed Student’s t-test was used for the statistical analysis. EAE is indicated in purple and DRhQ in blue.

**FIGURE 6:**
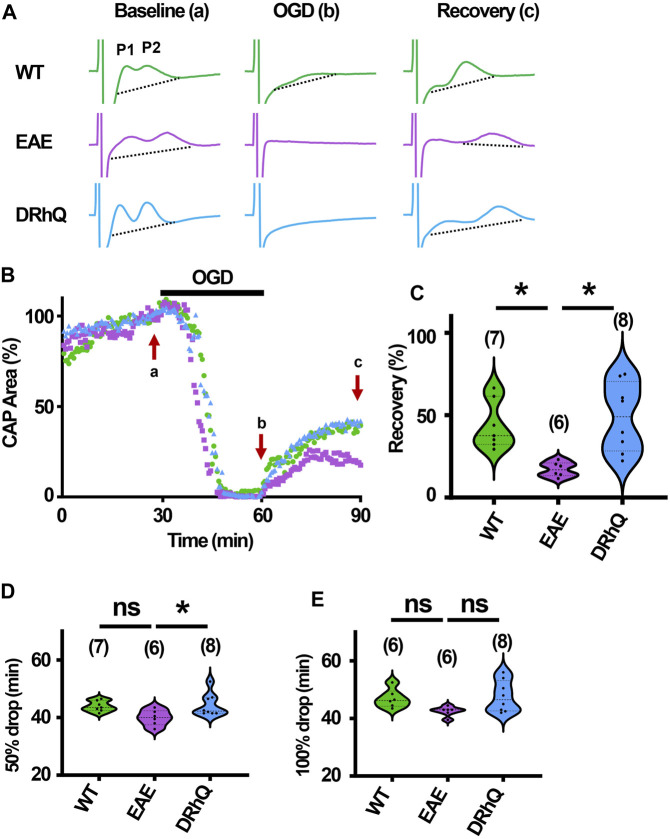
Axon function is highly vulnerable to ischemic injury. DRhQ improves axon function recovery. Wild type (WT) is indicated in green, EAE in purple, and DRhQ in blue. **(A)** Representative CAP traces from baseline (left), OGD (middle), and recovery (right), in WT (green), EAE + vehicle (purple), and EAE + DRhQ (light blue) mouse groups. Note that **(A)** representing myelinated axons and **(B)** representing unmyelinated axons are the two peaks observed in corpus callosum CAP recordings. **(B)** Time course displays CAP area changes when CC were exposed to 30 min of OGD. CAP area is quantified by measuring the area above the black dotted lines and the response and normalized to the last 10 min of baseline. **(C)** Vulnerability of white matter function is assessed as the CAP area recovery after OGD, normalized to baseline response. WT: 35.1 ± 4.3, EAE: 14.5 ± 2.1, DRhQ: 47.8 ± 6.6, ***p = 0.0008. **(D, E)** Time to 50% and 100% drop are measured as the time starting from the beginning of the protocol, where the evoked CAP area reached respectively 50% and 0% of the baseline. WT: 45.2 ± 0.7, EAE: 40.4 ± 1.5, DRhQ: 46.2 ± 1.1, **p = 0.0057. One-way ANOVA with a Bonferroni’s post-hoc correction was used for the statistical analysis and data is presented as mean ± SEM.

**FIGURE 7:**
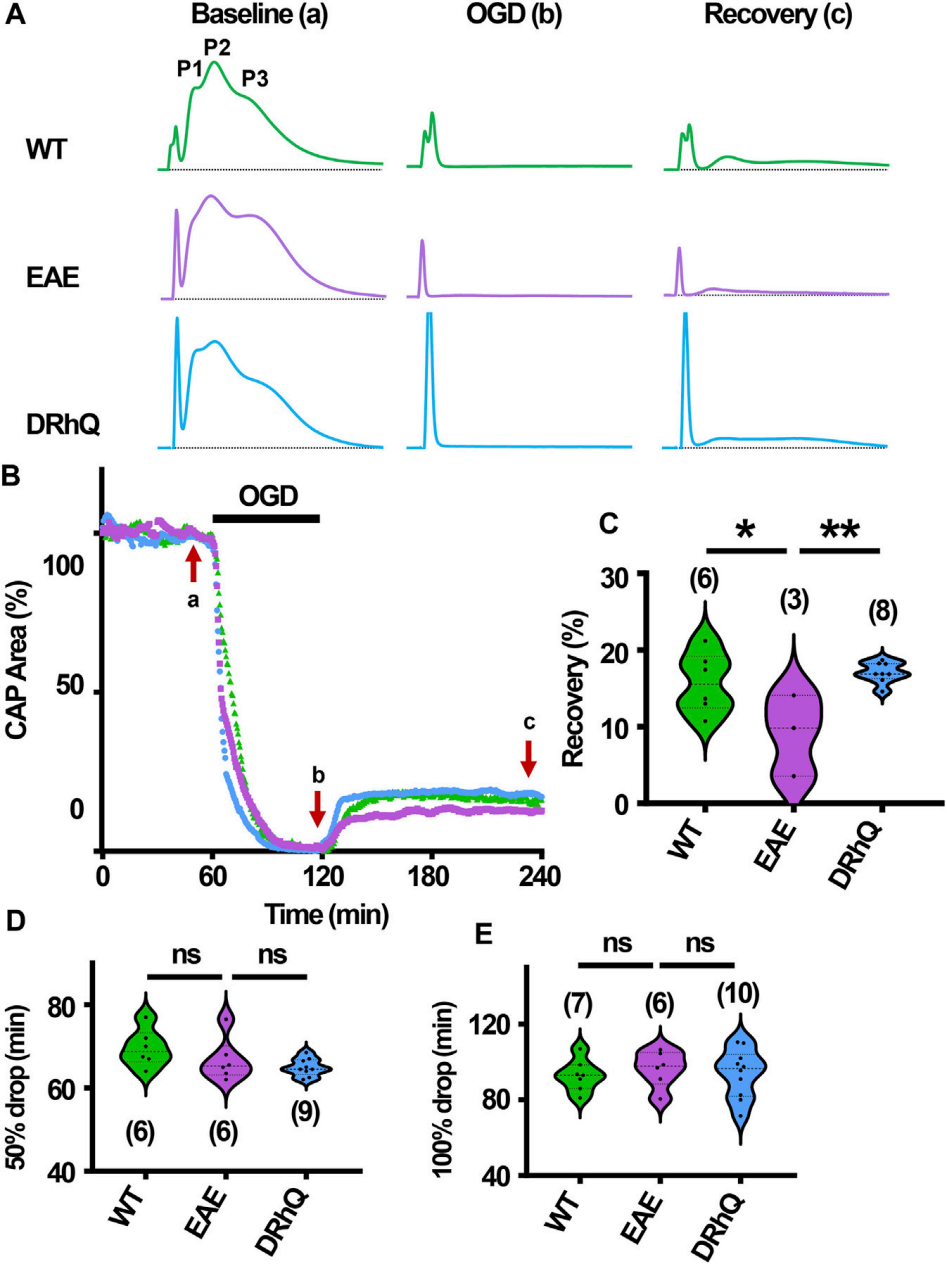
Axon function is highly vulnerable to ischemic injury. DRhQ improves axon function recovery. Wild type (WT) is indicated in green, EAE in purple, and DRhQ in blue. **(A)** Representative CAP traces from baseline (left), OGD (middle), and recovery (right), in WT (green), EAE + vehicle (purple), and EAE + DRhQ (light blue) mouse groups. Note that **(A)** representing myelinated axons, **(C)** representing unmyelinated axons, and **(B)** representing intermediate fibers, are the three peaks observed in optic nerves (MONs) CAP recordings. **(B)** Time course displays CAP area changes when MONs was exposed to 60 min of OGD. CAP area is quantified by measuring the area above the black dotted lines and the response and normalized to the last 10 min of baseline. **(C)** Vulnerability of white matter function is assessed as the CAP area recovery after OGD, normalized to baseline response. WT: 15.7 ± 1.6, EAE: 9.1 ± 3.0, DRhQ: 17.0 ± 0.5, **p = 0.0094. **(D, E)** Time to 50% and 100% drop are measured as the time starting from the beginning of the protocol, where the evoked CAP area reached respectively 50% and 0% of the baseline. WT: 69.6 ± 1.8, EAE: 66.7 ± 2.1, DRhQ: 64.9 ± 0.7, ns *p* = 0.1054. One-way ANOVA with a Bonferroni’s post-hoc correction was used for the statistical analysis and data is presented as mean ± SEM.

Function across the CC or MONs was quantified as the area under the evoked CAPs, using pClamp 10.3 software (Molecular Devices, United States). The CAP area represents the summation of all functional axons (Cummins et al., 1979; Stys et al., 1991). When recording in the CC, a typical CAP profile presents with 2 peaks; the first peak is the response of the fast-myelinated fibers, and the second represents the response of the slow lightly/unmyelinated fibers. Axon excitability was quantified by measuring the CAP area elicited at different varying stimulus intensities (1 mA, 0%–100% with 10% increments). CAP amplitude as a function of stimulus intensity was plotted to compare axon excitability among groups. Irreversible injury was measured by determining residual CAP area, normalized to the control CAP area, at least 2 h after the conclusion of OGD. Original data were normalized by setting the mean of initial baseline values (measured over 15 min) to a value of 100. Experimental results were pooled, averaged, and plotted against time. All data in Figure 1 through Figure 7 are presented as mean ± SEM (GraphPad Prism 9.3.1, GraphPad Software, Inc. La Jolla, CA). Two-tailed Student’s t-test (Figures 3, 5), two-way ANOVA (Figures 1, 2, 4, 6) and one-way ANOVA (Figures 2, 4, 6, 7) with a Bonferroni’s post-hoc correction were used for the statistical analysis. Statistical significance was set at *p* < 0.05 and indicated by an asterisk. The “n” values represent the number of brain slices or MONs recorded. Graphs and time courses were plotted with GraphPad Prism 9 and Microsoft Excel.

## Results

### Treatment with DRhQ decreases severity of acute EAE resulting in significantly improved clinical scores

After immunization, mice were assessed for signs of EAE according to the EAE score scale described in Methods. The period between immunization and EAE onset was of 11.4 days (±1.1) days for ‘EAE + vehicle’, and of 13.7 (±0.7) days for ‘EAE + DRhQ’ mice. Figure 1 shows that treatment with DRhQ (blue squares) or vehicle (violet circles) of immunized mice started after disease onset when the clinical EAE score was 2. After obtaining a clinical EAE score of 2, mice received 5 consecutive single daily injections of DRhQ or vehicle (Figure 1, red arrows).

Mice treated with vehicle (“EAE + vehicle”) showed progressively more severe clinical EAE scores, reaching a score of 5 only 2 days after receiving the first injection of vehicle, a level that was sustained through the end of the observation period. In contrast, treatment with DRhQ (“EAE + DRhQ”) instantly diminished progression of clinical EAE scores after the first day of injection (D1) compared to EAE + vehicle mice (EAE + vehicle, n = 5: 3.8 ± 0.1; EAE + DRhQ, n = 6: 2.5 ± 0.04, (***p = 0.0007, Two-way ANOVA, Bonferroni’s correction).

On D3, DRhQ treated animals showed a maximal EAE score of 2.8 (±0.1, n = 6). On D4, the last day of the injections, the course of EAE severity stabilized and started to go down on day six through day 10. The EAE scores of DRhQ treated animal were 1.5 on day 10, significantly lower than EAE + vehicle’s score on the same day (EAE + vehicle (n = 4): 4.8 ± 0.1, EAE + DRhQ (n = 6): 1.5 ± 0.2, ****p < 0.0001, Two-way ANOVA, Bonferroni’s correction). These results suggest that DRhQ efficiently modifies EAE severity by arresting and then reversing disease progression in adult mice.

### DRhQ restores axon function properties in corpus callosum impaired with EAE

Corpus callosum (CC), the largest compact WM tract of the human brain involved in interhemispheric transfer, is often damaged during multiple sclerosis (MS). Lesions in the CC are important radiological clues to the diagnosis of MS. Therefore, in this study, we directly assessed axonal excitability and axonal conduction properties using coronal slices containing CC. Our previous studies have established coronal CC slices as a reliable and reproducible model to assess axon function and WM architecture (Tekkok and Goldberg, 2001; Tekkok and Ransom, 2004; Bastian et al., 2022). To monitor the functional integrity of axons, we recorded CAPs across the CC elicited by a bipolar stimulation electrode. The CC tract is composed of myelinated (30%) and unmyelinated (70%) fibers (Seggie and Berry, 1972; Sturrock, 1980; Preston et al., 1983; Hildebrand et al., 1993; Nunez et al., 2000; Tekkok and Ransom, 2004) and subsequently CAPs typically exhibit two peaks (P); P1 for myelinated axons with a faster conduction time, and P2 for unmyelinated axons with a slower conduction time (Figure 2A). Recording configuration was adjusted to elicit a CAP with two distinct peaks; P1 and P2 to represent myelinated and unmyelinated axons. If only one peak is visible, the electrodes were adjusted to elicit CAP with two visible peaks. To determine the impact of EAE and DRhQ treatment on axon conduction properties, CAPs were recorded for 30 min under baseline conditions. CAP area (Figure 2A, depicted in gray shaded area) is a measure of all stimulated axon response that is quantified by integrating action potential amplitude (mV) as a function of spatiotemporal summation measured as CAP duration (ms) (Figure 2A, black dotted arrow). In CC slices obtained from wild type, EAE and DRhQ treated mice, the CAP area values remained similar between WT and EAE, EAE and DRhQ, while compared to WT group, CAP area values of DRhQ group were increased (Figure 2B). Interestingly, despite similar CAP area, CAP duration (ms, Figure 2C), increased in EAE group compared to WT and treated groups. These results suggest slower spatiotemporal summation of action potentials after EAE due to loss of myelin and axon damage (see Figure 3A). DRhQ treatment preserved axon structure and myelin (Figure 3A) and improved axon function leading to shortened CAP duration (Figure 2C). On the other hand, P1 in EAE group was drastically decreased but restored with DRhQ (Figure 2D, in mV), while the amplitude of the second peak remained unchanged between the groups (Figure 2E) suggesting the main target for EAE was myelinated axons as opposed to unmyelinated fibers. Quantification showed increased time to P1 latency (depicted in Figure 2A as dotted lines from green stimulus artefact to P1 in pink and to P2 in blue) in EAE compared to WT which were improved with DRhQ treatment (Figure 2F). P2 representing slower conducting fibers displayed a similar reduction in conduction velocity after EAE which was restored with DRhQ treatment (Figure 2G). Consistent with the altered axonal conduction properties, EAE caused an unexpected increase in axon excitability at certain stimulus intensity range (Figure 2H) which was attenuated following DRhQ treatment.

### DRhQ alleviates EAE-mediated alterations on microglia, astrocytes, axons, and myelin

To examine the mechanisms by which DRhQ improves axon function after EAE, we studied the targets of EAE on the cellular components of CC. WM is composed of axons, myelin, oligodendrocytes, microglia, and astrocytes. Using cell-specific antibodies, we assessed Iba (+) microglia, GFAP (+) astrocytes, NF160/200 (+) axons and MBP (+) myelin (Figure 3A) by quantifying fluorescent density in immunolabeled sections from EAE and DRhQ treated sections (Figure 3B). DRhQ (blue violins) effectively reduced widespread upregulation in microglia and astrocyte labeling intensity (p(s) < 0.01, unpaired t-test) while improving axonal and myelin labeling in CC slices (p(s) < 0.05 unpaired t-test) (Figure 3). We suggest that a reduction in microglial and astrocyte labeling intensity reflects an attenuation in their activation and EAE-induced immune response. The enhancement of axon and myelin labeling is in agreement with restored axon function characteristics with DRhQ treatment (Figure 1).

### DRhQ restores axon function properties in optic nerve impaired with EAE

Optic neuritis, an inflammatory injury to the myelin sheath of the optic nerve that is characterized with pain and vision loss, commonly occurs in MS patients (Chan, 2002; Arnold, 2005; Kale, 2016). We aimed to directly assess how EAE impacts fully myelinated axon function in the optic nerve, and whether anti-inflammatory effects of DRhQ might have protective benefits on the visual tract. Mouse optic nerves (MONs) obtained from WT, EAE and DRhQ mice were placed in between two suction electrodes delivering stimulation to the retinal side and recording evoked responses from the chiasmal side. This recording configuration allows stimulation of all axons in the MONs such that a CAP represents all axons thereby providing information on the number, speed, and spatiotemporal summation of axonal function in a nerve bundle. Recordings from MONs typically exhibited CAPs with three peaks (P); P1 (Figure 4A, depicted with pink line) for thicker axons with thicker myelin with a fastest conduction time, P2 (Figure 4A, depicted by an orange line) for intermediate size and myelin content fibers, and P3 (Figure 4A, depicted in blue line) for smaller diameter and lightly myelinated axons with the slowest conduction time. Axonal conduction properties in MONs showed a similar change to CC such that CAP area remained (Figure 4A, depicted by a gray shaded area) unchanged with EAE or DRhQ treatment (Figure 4B) while CAP duration (Figure 4A, depicted by a black arrow) significantly increased in the EAE group while treatment with DRhQ effectively improved spatiotemporal summation of CAPs (Figure 4C). P1 amplitude (Figure 4A, depicted in pink arrow) representing the fastest conducting myelinated axons was suppressed by 40%, but P2 and P3 amplitudes were conserved (Figures 4D–F). However, the latency of all peaks P1, P2, P3 (Figure 4A, depicted in purple, dark yellow, and blue) considerably became prolonged with EAE suggesting a slowing of conduction in all axons, which was resolved with DRhQ (Figures 4G–I) This subsequently resulted in prolonged CAP duration, also restored with DRhQ (Figure 4C). Measuring CAP area over a range of stimulus intensities identified minimal changes in axon excitability with a lower excitability of EAE axons at two different stimulation intensities compared to WT, while no differences were observed between WT and DRhQ, or EAE and DRhQ (Figure 4J).

### DRhQ alleviates EAE-mediated alterations on astrocytes, oligodendrocytes, axons, and myelin in optic nerves

To confirm that structural and cellular changes occurred in MON slices, we immunolabeled astrocytes with GFAP, oligodendrocytes with Olig-2, axonal neurofilaments with NF160/200 and myelin with MBP in optic nerve sections (Figure 5A) and compared labeling intensity and structural changes between EAE and DRhQ treated groups (Figure 5B). Similar to CC slices, DRhQ attenuated GFAP labeling intensity suggesting a downregulation of astrocyte activation (*p* < 0.0001, unpaired Student’s t-test) (Figures 5A, B). DRhQ treatment rescued Olig-2 (+) oligodendrocyte cell death caused by EAE quantified by cell count per 1.0 × 104 µm^2^ (*p* < 0.0001, unpaired Student’s t-test) (Figures 5A, B). DRhQ treatment also improved axon and myelin labeling which was diminished with EAE (*p* < 0.0001, unpaired Student’s t-test) (Figures 5A, B). These results suggest that DRhQ dampens EAE-induced astrocytes activation and preserves axon function by protecting axons, oligodendrocytes and myelin in optic nerves.

### EAE renders axons more vulnerable to subsequent ischemic injury

The role of inflammation in ischemic WM disease is increasingly documented and the risk for cerebral ischemia in people with MS increases (Tseng et al., 2015; Hong et al., 2019; Doskas et al., 2022). Therefore, we hypothesized that EAE may render WM more vulnerable to a subsequent ischemic injury. Note the distinct, sharper rapidly rising P1 and P2 in CAPs elicited from WT MONs (Figure 6A, sample traces depicted in green) which become less distinguished dull and slower to rise with EAE (Figure 6A, sample traces depicted in purple). Treatment with DRhQ impressively restored the CAP shape and peaks (Figure 6A, sample traces in blue). After at least 30 min of stable baseline, CC slices were metabolically challenged with oxygen glucose deprivation (OGD) for 30 min.

At end of the OGD, CAPs were lost in EAE + vehicle and EAE + DRhQ groups, while in 1/7 slices a minimal CAP in WT group remained at the end of the OGD (Figure 6A). During OGD, CAP area of EAE + vehicle mice, tended to drop faster than WT and EAE + DRhQ treated groups (Figures 6B, D, E). CC slices from EAE + vehicle mice recovered less than WT and EAE + DRhQ treated groups (Figures 6B, C). This suggests that treatment with DRhQ improves WM resilience to a metabolic challenge in EAE mice.

### DRhQ treatment improves axon function recovery after ischemia

After 60 min of stable baseline, MONs were exposed to OGD for 60 min (Figure 7B). During OGD, CAP area dropped at a similar rate among the three groups (Figures 7B, D, E) At end of the OGD, CAPs were completely lost (Figures 7A, B). MONs from EAE + vehicle mice recovered less compared to WT and EAE + DRhQ treated MONs (Figures 7B, C). DRhQ treatment may improve axon function recovery post ischemic injury.

## Discussion

Demyelinating lesions and optic neuritis are hallmarks of MS (Chan, 2002; Arnold, 2005; Breij et al., 2008; Kutzelnigg and Lassmann, 2014; Kale, 2016), and current treatments are disease-modulating drugs and for the vast majority, only efficient on the RRMS types, with little benefit on progressive MS. Moreover, current, therapies for optic neuritis are subject to controversy. There is a need for treatments that cover all aspects and pathologies of MS. EAE is a commonly used MS mouse model to develop and validate disease-modulating therapies. Previous studies have focused on the RTL1000 and DRα1-hMOG-35-55 constructs that showed benefits in clinical, behavioral, and histological improvement in EAE mice (Vandenbark et al., 2003; Meza-Romero et al., 2014; Benedek et al., 2015; Benedek et al., 2017; Yang et al., 2017; Meza-Romero et al., 2019; Vandenbark et al., 2019; Vandenbark et al., 2021; Vandenbark et al., 2022; Offner et al., 2023). Of note, the MOG-35-55 peptide was initially included in previous RTL1000 and DRα1 constructs to mimic a soluble T cell receptor ligand that could selectively target and block MOG-35-55 specific T cells during EAE induction (Vandenbark et al., 2022). Therefore, one possible mechanism of these constructs would be targeting DR2/1501 restricted MOG-35-55-specific TCRs and thus RTL1000 construct was administered to pre-selected DR2/1501 MS recipients (which represent ∼50% of the MS population) in a Phase 1 clinical trial carried out in 2007–2009 showing safety and tolerability (Yadav et al., 2012). However, we have identified that RTL1000 construct not only binds to CD74 but also competitively blocks MIF binding and downstream signaling (Meza-Romero et al., 2014). To make the inhibitory construct non-MHC restricted and more generally useful for blocking MIF effects, we removed the DR2/1501 β1 domain and made an L50Q substitution that increased binding affinity to CD74. This 3rd generation construct, called DRhQ, was no longer DR2 restricted, had reduced effects on MOG-35-55 specific T cells, but retained the ability to block MIF effects in a number of different inflammatory conditions (stroke, methamphetamine abuse, and TBI) (Benedek et al., 2017; Yang et al., 2017; Meza-Romero et al., 2019; Vandenbark et al., 2019) in different mouse and rat strains without MHC restriction. Moreover, in the absence of the DR2 β1 domain, DRhQ now functions uniquely as a CD74 inhibitor rather than a TCR ligand, with MOG-35-55 providing stability to the construct rather than selectively targeting the TCR of MOG-35-55 specific T cells.

Our objective using the third generation DRhQ construct that possesses enhanced CD74 binding affinity demonstrates that treatment after onset of clinical signs of EAE directly regulates axon conduction properties by conserving oligodendrocytes, myelin and axonal neurofilaments and attenuating astrocyte and microglia activation. Of mechanistic importance, all of these CNS cell types express CD74 and can be negatively affected by MIF/CD74-driven inflammation (Su et al., 2017; Yang et al., 2017; Vandenbark et al., 2019), but countered by DRhQ blockade of CD74 activation. In our study, we utilized two distinct *in vitro* WM tracts and provided direct evidence that DRhQ has “repair” capacity, for it reversed EAE damage. Hence, we propose DRhQ as an effective therapeutic with possible clinical applications for patients with MS as well as those with other MIF/CD74 driven CNS conditions, including ischemic injury, traumatic brain injury, methamphetamine use disorder and Alzheimer’s disease.

MS is characterized by demyelination in grey and WM, and multifocal inflammation (Prineas et al., 2001; Dutta and Trapp, 2007; Trapp and Nave, 2008; Dutta and Trapp, 2014), with oligodendrocyte loss. Consistent with EAE induction, C57BL/6 mice (Glatigny and Bettelli, 2018; Constantinescu et al., 2011; t Hart et al., 2011) exhibited a progressively deteriorating neurological performance correlated with a prominent myelin and oligodendrocyte loss. A prominent delay in each peak of CAPs recorded from CC or MON axons was the most direct evidence of myelin loss and the resultant conduction delay. Prolonged CAP duration was another electrical signature of myelin loss implying a slower spatiotemporal summation of individual action potentials. Consequently, these findings infer alterations in nodal-paranodal structures, which are indeed reported to precede the onset of demyelination (Stojic et al., 2018). However, DRhQ, even when administered after the disease onset, effectively restored peak latencies in both WM tracts suggesting that DRhQ can repair myelin disruption and restore axon conduction. The myelin repair by DRhQ correlated with an increase in oligodendrocyte numbers. Indeed, cycles of demyelination and remyelination are characteristic of the RRMS type MS (Chang et al., 2012) with activation of oligodendrocytes progenitor cells differentiation into mature oligodendrocytes (Sachs et al., 2014) to maintain myelin production and axon integrity. It is yet not clear, however, whether the rescued oligodendrocytes with DRhQ treatment are newly differentiated oligodendrocytes, or if DRhQ interrupted oligodendrocyte death due to EAE pathology.

Consistent with axonal damage (De Stefano et al., 2002; Filippi et al., 2003; Pascual-Lozano et al., 2007; Trapp and Nave, 2008), a diffuse loss of NF160/200 (+) neurofilament immunoreactivity in axons was evident and accompanied myelin loss. The neurofilament damage was reflected as a prominent decrease in the first peak amplitudes of CAPs recorded in both WM tracts. The first peaks in CAPs represent the functional sum of myelinated axons while other peaks are sum of action potentials generated by unmyelinated or lightly myelinated axons. It is plausible that axons that become demyelinated with EAE may contribute to the second or third peaks hindering a drop in the amplitude of these peaks. Because neurofilaments are neuron specific cytoskeletal proteins (Yuan et al., 2012) that can be released into extracellular fluid following axon injury (Petzold, 2005), in MS clinical settings a growing body of evidence support that neurofilament levels in cerebrospinal fluid (CSF) and serum are a reliable indicator of prognosis and treatment response and facilitate individual treatment decisions (Salzer et al., 2010; Teunissen and Khalil, 2012; Bittner et al., 2021). Therefore, serum or CSF neurofilament levels are practical biomarkers used for MS patients.

Myelin loss and axonal damage due to EAE in CC and MONs are accompanied by diffuse activation of Iba (+) microglia and GFAP (+) astrocytes. There are two forms of axonal injury during MS; one is demyelination and the other is inflammation. Inflammation is closely related to activated microglia and astrocytes and MS lesions show activation of astrocytes and microglia. Microglia are resident phagocytes of the CNS and are abundantly present in MS lesions, but their role -whether protective or injurious-has been debated (Fu et al., 2014). Microglia and monocyte activation can strongly influence myelin regeneration by cleaning debris or releasing more cytokines to exacerbate inflammation (Lloyd et al., 2017). Astrocytes are a principal member of the MS plaque and can also enhance inflammation by releasing effector molecules (Smith and Sommer, 1992; Ludwin et al., 2016), but they may also limit damage by taking up glutamate, providing metabolic stability and maintaining the blood-brain barrier (Newcombe et al., 2008). The functional benefits of the DRhQ correlated with attenuation of astrocyte and microglia activation in CC and MONs, suggesting more of an injurious role for their activated form in EAE. Such activation may well be due to increased levels of MIF/DDT binding to CD74 expressed by astrocytes and microglia within the CNS, a process that could be directly interrupted by DRhQ blockade of CD74 signaling.

With many aspects of MS pathology, hyperexcitability can be observed in patients as positive symptoms such as paranesthesia, tonic seizures, or Lhermitte’s sign (electric shock like condition) (Sakurai and Kanazawa, 1999; Sa, 2012). Consistent with these reports, EAE pathology caused an increased axon excitability in CC, with a higher axonal response in EAE mice compared to control conditions. Yet, in MONs, axonal excitability changes were less pronounced and EAE mice displayed a lower excitability compared to WT, with axonal responses only at 30%–40% of the maximum intensity. In the EAE model, damage to the nodes of Ranvier are described in WM, with alteration of sodium channel expression at the nodal region (Craner et al., 2003; Craner et al., 2004; Lubetzki et al., 2020), as well as an increased length of the nodal region (Fu et al., 2011) leading to changes in neuronal excitability (Ellwardt et al., 2018). Sodium channels are crucial for the action potential generation (Guy and Seetharamulu, 1986). Based on these findings, the hyperexcitability observed in CC, and to a lesser extent in MONs, could be the result of these alterations, and treatment with DRhQ alleviated these excitability alterations. Besides its protective effect on myelin, it would be of great interest to further explore DRhQ effects on excitability alteration in MS pathology.

People with MS have an increased risk of developing any type of stroke (Hong et al., 2019). Stroke, a leading cause of death and disability, is the third most common cause of disease worldwide. Patients with MS may suffer from more severe symptoms when they experience an ischemic attack. Expectedly, axon function recovered less in EAE mice when exposed to OGD. Mechanisms of ischemia follow a spatiotemporal course in WM triggered by Na + overload, leading to excitotoxicity mediated by AMPA/kainate receptor overactivation followed by mitochondrial collapse and oxidative injury. Glutamate is released via the reversal of the glutamate transporter 1 (GLT-1), due to increased Na + levels, resulting in excitotoxicity and irreversible ischemic injury (Tekkok et al., 2007). Excitotoxicity is proposed to cause neurodegeneration in MS (Gonsette, 2008). There is evidence of altered glutamate metabolism in WM of MS patients (Srinivasan et al., 2005). In brain tissues from MS patients, transporter GLT-1 is found to be downregulated (Vercellino et al., 2007) — suggesting an altered glutamate reuptake in the synaptic cleft—in the presence of activated microglia and myelin and neuronal damage (Vercellino et al., 2007). Moreover, blocking the AMPA/Kainate receptor alleviates the pathology in EAE mice without modifying the inflammation in lesions (Pitt et al., 2000), suggesting that the proposed excitotoxicity in MS could be mediated by an upregulation of AMPA/kainate receptors, and involved in increased vulnerability of EAE axon function to ischemia.

Axon function in CC is more resilient to OGD followed by better recovery compared to MONs. This could be due to the combination of myelinated and unmyelinated axons found in CC as opposed to fully myelinated axons in MON. Interestingly, the rate of axon function loss during OGD remained the same between the control and EAE groups despite poorer recovery after OGD in EAE mice. This negates the idea that axons in the EAE model experience energy deprivation (Redford et al., 1997; Smith et al., 2001; Dutta et al., 2006). Axons are independent of their neuronal cell bodies and intimately rely on local mitochondria and ATP generation (Chen and Sheng, 2013; Chamberlain and Sheng, 2019). In MS, sustained inflammation leads to production of mitochondria with respiratory chain deficiency and mitochondrial transport, creating an energy imbalance contributing to enhanced oxidative injury (Witte et al., 2014). Future experiments will directly test mitochondrial dynamics in EAE and whether protective effects of DRhQ are mediated through mitochondrial regulation in WM.

One limitation in our study is that the experiments were conducted in male mice while MS predominantly affects women compared to men (Alonso and Hernan, 2008). EAE male mice display a lower CNS infiltration and demyelination compared to female mice, while the proinflammatory response of immune cells is higher in males compared to females. Therefore, overall, in the EAE mouse model, disease severity is the same in both sexes (Wiedrick et al., 2021) and DRhQ equally decreases the severity of the disease in male and in female EAE mice (Vandenbark et al., 2022). However, our future follow up studies will investigate if there are any sex-dependent effects of EAE and the subsequent DRhQ benefits.

It is of note that Fingolimod, which is a sphingosine I-phosphate receptor modulator in use to treat the relapsing forms of multiple sclerosis (MS), including clinically isolated syndrome, relapsing-remitting disease, and active secondary progressive disease to slow some disabling effects and decrease the number of relapses of the disease, has failed to show a comparable benefit for axon function in MONs (Baltan et al., unpublished). Therefore, the consistent “repair” capacity of DRhQ in the EAE model is exceptional and warrants further studies to validate its benefit in MS patients.

## Conclusion

In summary, our results show that EAE pathology alters axon function properties and renders WM highly vulnerable to ischemic injury in two WM tracts. DRhQ administered after EAE induction restored WM function and attenuated vulnerability to ischemic injury. Our study implies a therapeutic potential of DRhQ that may provide future treatment for MS patients.

## Figures and Tables

**Table:** 

**APC**	Antigen-presenting cells
**aCSF**	Artificial Cerebrospinal Fluid
**BBB**	Blood–brain barrier
**CD74, II**	Cluster of Differentiation 74
**CC**	Corpus callosum
**CAP**	Compound action potential
**CFA**	Complete Freund’s Adjuvant
**CNS**	Central nervous system (CNS)
**D**	Day
**D-DT**	D-dopachrome tautomerase
**DRhQ**	DRα1-MOG-35-55
**EAE**	Experimental autoimmune encephalomyelitis
**GFAP**	Glial Fibrillary Acidic Protein
**IACUC**	Institutional Animal Care and Use Committee
**MAPK**	Mitogen-activated protein kinase
**MBP**	Myelin Basic Protein
**MOG**	Myelin oligodendrocyte glycoprotein
**MONs**	Mouse optic nerves
**MS**	Multiple sclerosis
**MIF**	Macrophage inhibitory factor
**MHC**	Major histocompatibility complex
**NF**	Neurofilament
**OGD**	Oxygen glucose deprivation
**P**	Peak
**PPMS**	Primary progressive MS
**PRMS**	Progressive-remitting MS
**Ptx**	Pertussis toxin
**RRMS**	Relapsing-remitting MS
**RTL**	Recombinant T-cell receptor ligand
**SPMS**	Secondary progressive MS
**WT**	Wild type

## Data Availability

The raw data supporting the conclusion of this article will be made available by the authors, without undue reservation.

## References

[B1] Alonso A. Hernan M. A. (2008). Temporal trends in the incidence of multiple sclerosis: a systematic review. Neurology 71 (2), 129–135. 10.1212/01.wnl.0000316802.35974.34 18606967 PMC4109189

[B2] Arnold A. C. (2005). Evolving management of optic neuritis and multiple sclerosis. Am. J. Ophthalmol. 139 (6), 1101–1108. 10.1016/j.ajo.2005.01.031 15953446

[B3] Bastian C. Zerimech S. Nguyen H. Doherty C. Franke C. Faris A. (2022). Aging astrocytes metabolically support aging axon function by proficiently regulating astrocyte-neuron lactate shuttle. Exp. Neurol. 357, 114173. 10.1016/j.expneurol.2022.114173 35863500 PMC11218845

[B4] Benedek G. Meza-Romero R. Jordan K. Keenlyside L. Offner H. Vandenbark A. A. (2015). HLA-DRα1-mMOG-35-55 treatment of experimental autoimmune encephalomyelitis reduces CNS inflammation, enhances M2 macrophage frequency, and promotes neuroprotection. J. Neuroinflammation 12, 123. 10.1186/s12974-015-0342-4 26104759 PMC4481122

[B5] Benedek G. Vandenbark A. A. Alkayed N. J. Offner H. (2017). Partial MHC class II constructs as novel immunomodulatory therapy for stroke. Neurochem. Int. 107, 138–147. 10.1016/j.neuint.2016.10.007 27773790 PMC5411346

[B6] Bittner S. Oh J. Havrdova E. K. Tintore M. Zipp F. (2021). The potential of serum neurofilament as biomarker for multiple sclerosis. Brain 144 (10), 2954–2963. 10.1093/brain/awab241 34180982 PMC8634125

[B7] Breij E. C. Brink B. P. Veerhuis R. van den Berg C. Vloet R. Yan R. (2008). Homogeneity of active demyelinating lesions in established multiple sclerosis. Ann. Neurol. 63 (1), 16–25. 10.1002/ana.21311 18232012

[B8] Chamberlain K. A. Sheng Z. H. (2019). Mechanisms for the maintenance and regulation of axonal energy supply. J. Neurosci. Res. 97 (8), 897–913. 10.1002/jnr.24411 30883896 PMC6565461

[B9] Chan J. W. (2002). Optic neuritis in multiple sclerosis. Ocul. Immunol. Inflamm. 10 (3), 161–186. 10.1076/ocii.10.3.161.15603 12789593

[B10] Chang A. Staugaitis S. M. Dutta R. Batt C. E. Easley K. E. Chomyk A. M. (2012). Cortical remyelination: a new target for repair therapies in multiple sclerosis. Ann. Neurol. 72 (6), 918–926. 10.1002/ana.23693 23076662 PMC3535551

[B11] Chen Y. Sheng Z. H. (2013). Kinesin-1-syntaphilin coupling mediates activity-dependent regulation of axonal mitochondrial transport. J. Cell Biol. 202 (2), 351–364. 10.1083/jcb.201302040 23857772 PMC3718985

[B12] Constantinescu C. S. Farooqi N. O'Brien K. Gran B. (2011). Experimental autoimmune encephalomyelitis (EAE) as a model for multiple sclerosis (MS). Br. J. Pharmacol. 164 (4), 1079–1106. 10.1111/j.1476-5381.2011.01302.x 21371012 PMC3229753

[B13] Craner M. J. Hains B. C. Lo A. C. Black J. A. Waxman S. G. (2004). Co-localization of sodium channel Nav1.6 and the sodium-calcium exchanger at sites of axonal injury in the spinal cord in EAE. Brain 127 (2), 294–303. 10.1093/brain/awh032 14662515

[B14] Craner M. J. Lo A. C. Black J. A. Waxman S. G. (2003). Abnormal sodium channel distribution in optic nerve axons in a model of inflammatory demyelination. Brain 126 (7), 1552–1561. 10.1093/brain/awg153 12805113

[B15] Cummins K. L. Perkel D. H. Dorfman L. J. (1979). Nerve fiber conduction-velocity distributions. I. Estimation based on the single-fiber and compound action potentials. Electroencephalogr. Clin. Neurophysiol. 46 (6), 634–646. 10.1016/0013-4694(79)90101-9 87308

[B16] De Stefano N. Narayanan S. Francis S. J. Smith S. Mortilla M. Tartaglia M. C. (2002). Diffuse axonal and tissue injury in patients with multiple sclerosis with low cerebral lesion load and no disability. Arch. Neurol. 59 (10), 1565–1571. 10.1001/archneur.59.10.1565 12374493

[B17] Doskas T. Vavougios G. D. Karampetsou P. Kormas C. Synadinakis E. Stavrogianni K. (2022). Neurocognitive impairment and social cognition in multiple sclerosis. Int. J. Neurosci. 132 (12), 1229–1244. 10.1080/00207454.2021.1879066 33527857

[B18] Dutta R. McDonough J. Yin X. Peterson J. Chang A. Torres T. (2006). Mitochondrial dysfunction as a cause of axonal degeneration in multiple sclerosis patients. Ann. Neurol. 59 (3), 478–489. 10.1002/ana.20736 16392116

[B19] Dutta R. Trapp B. D. (2007). Pathogenesis of axonal and neuronal damage in multiple sclerosis. Neurology 68 (22), S22–S31. 10.1212/01.wnl.0000275229.13012.32 17548565

[B20] Dutta R. Trapp B. D. (2014). Relapsing and progressive forms of multiple sclerosis: insights from pathology. Curr. Opin. Neurol. 27 (3), 271–278. 10.1097/WCO.0000000000000094 24722325 PMC4132635

[B21] Ellwardt E. Pramanik G. Luchtman D. Novkovic T. Jubal E. R. Vogt J. (2018). Maladaptive cortical hyperactivity upon recovery from experimental autoimmune encephalomyelitis. Nat. Neurosci. 21 (10), 1392–1403. 10.1038/s41593-018-0193-2 30258239

[B22] Filippi M. Bozzali M. Rovaris M. Gonen O. Kesavadas C. Ghezzi A. (2003). Evidence for widespread axonal damage at the earliest clinical stage of multiple sclerosis. Brain 126 (2), 433–437. 10.1093/brain/awg038 12538409

[B23] Fu R. Shen Q. Xu P. Luo J. J. Tang Y. (2014). Phagocytosis of microglia in the central nervous system diseases. Mol. Neurobiol. 49 (3), 1422–1434. 10.1007/s12035-013-8620-6 24395130 PMC4012154

[B24] Fu Y. Frederick T. J. Huff T. B. Goings G. E. Miller S. D. Cheng J. X. (2011). Paranodal myelin retraction in relapsing experimental autoimmune encephalomyelitis visualized by coherent anti-Stokes Raman scattering microscopy. J. Biomed. Opt. 16 (10), 106006. 10.1117/1.3638180 22029353 PMC3206924

[B25] Glatigny S. Bettelli E. (2018). Experimental autoimmune encephalomyelitis (EAE) as animal models of multiple sclerosis (MS). Cold Spring Harb. Perspect. Med. 8 (11), a028977. 10.1101/cshperspect.a028977 29311122 PMC6211376

[B26] Goldenberg M. M. (2012). Multiple sclerosis review. P T 37 (3), 175–184. 22605909 PMC3351877

[B27] Gonsette R. E. (2008). Oxidative stress and excitotoxicity: A therapeutic issue in multiple sclerosis? Mult. Scler. 14 (1), 22–34. 10.1177/1352458507080111 17881394

[B28] Guy H. R. Seetharamulu P. (1986). Molecular model of the action potential sodium channel. Proc. Natl. Acad. Sci. U. S. A. 83 (2), 508–512. 10.1073/pnas.83.2.508 2417247 PMC322889

[B29] Hildebrand C. Remahl S. Persson H. Bjartmar C. (1993). Myelinated nerve fibres in the CNS. Prog. Neurobiol. 40 (3), 319–384. 10.1016/0301-0082(93)90015-k 8441812

[B30] Hong Y. Tang H. R. Ma M. Chen N. Xie X. He L. (2019). Multiple sclerosis and stroke: a systematic review and meta-analysis. BMC Neurol. 19 (1), 139. 10.1186/s12883-019-1366-7 31234793 PMC6591845

[B31] Kale N. (2016). Optic neuritis as an early sign of multiple sclerosis. Eye Brain 8, 195–202. 10.2147/EB.S54131 28539814 PMC5398757

[B32] Kutzelnigg A. Lassmann H. (2014). Pathology of multiple sclerosis and related inflammatory demyelinating diseases. Handb. Clin. Neurol. 122, 15–58. 10.1016/B978-0-444-52001-2.00002-9 24507512

[B33] Lloyd A. F. Davies C. L. Miron V. E. (2017). Microglia: origins, homeostasis, and roles in myelin repair. Curr. Opin. Neurobiol. 47, 113–120. 10.1016/j.conb.2017.10.001 29073528

[B34] Lubetzki C. Sol-Foulon N. Desmazieres A. (2020). Nodes of Ranvier during development and repair in the CNS. Nat. Rev. Neurol. 16 (8), 426–439. 10.1038/s41582-020-0375-x 32651566

[B35] Ludwin S. K. Rao V. Moore C. S. Antel J. P. (2016). Astrocytes in multiple sclerosis. Mult. Scler. 22 (9), 1114–1124. 10.1177/1352458516643396 27207458

[B36] Meza-Romero R. Benedek G. Gerstner G. Kent G. Nguyen H. Offner H. (2019). Increased CD74 binding and EAE treatment efficacy of a modified DRα1 molecular construct. Metab. Brain Dis. 34 (1), 153–164. 10.1007/s11011-018-0331-2 30353480 PMC6364671

[B37] Meza-Romero R. Benedek G. Yu X. Mooney J. L. Dahan R. Duvshani N. (2014). HLA-DRα1 constructs block CD74 expression and MIF effects in experimental autoimmune encephalomyelitis. J. Immunol. 192 (9), 4164–4173. 10.4049/jimmunol.1303118 24683185 PMC4028955

[B38] Munoz J. J. Arai H. Bergman R. K. Sadowski P. L. (1981). Biological activities of crystalline pertussigen from Bordetella pertussis. Infect. Immun. 33 (3), 820–826. 10.1128/IAI.33.3.820-826.1981 6269999 PMC350785

[B39] Newcombe J. Uddin A. Dove R. Patel B. Turski L. Nishizawa Y. (2008). Glutamate receptor expression in multiple sclerosis lesions. Brain Pathol. 18 (1), 52–61. 10.1111/j.1750-3639.2007.00101.x 17924980 PMC8095601

[B40] Noubade R. Saligrama N. Spach K. Del Rio R. Blankenhorn E. P. Kantidakis T. (2008). Autoimmune disease-associated histamine receptor H1 alleles exhibit differential protein trafficking and cell surface expression. J. Immunol. 180 (11), 7471–7479. 10.4049/jimmunol.180.11.7471 18490747 PMC2543130

[B41] Nunez J. L. Nelson J. Pych J. C. Kim J. H. Juraska J. M. (2000). Myelination in the splenium of the corpus callosum in adult male and female rats. Brain Res. Dev. Brain Res. 120 (1), 87–90. 10.1016/s0165-3806(99)00193-5 10727734

[B42] Offner H. Lockwood D. Meza-Romero R. Vandenbark A. A. (2023). PD-L1 is required for estrogen-induced protection against severe EAE in IL-10 deficient mice(1). Metab. Brain Dis. 38 (2), 589–599. 10.1007/s11011-022-01129-8 36454506 PMC9976593

[B43] Pascual-Lozano A. M. Martinez-Bisbal M. C. Bosca-Blasco I. Valero-Merino C. Coret-Ferrer F. Marti-Bonmati L. (2007). Total brain T2-hyperintense lesion-volume and the axonal damage in the normal-appearing white matter of brainstem in early lapsing-remitting multiple sclerosis. Rev. Neurol. 45 (8), 468–473. 10.33588/rn.4508.2007087 17948212

[B44] Petzold A. (2005). Neurofilament phosphoforms: surrogate markers for axonal injury, degeneration and loss. J. Neurol. Sci. 233 (1-2), 183–198. 10.1016/j.jns.2005.03.015 15896809

[B45] Pitt D. Werner P. Raine C. S. (2000). Glutamate excitotoxicity in a model of multiple sclerosis. Nat. Med. 6 (1), 67–70. 10.1038/71555 10613826

[B46] Preston R. J. Waxman S. G. Kocsis J. D. (1983). Effects of 4-aminopyridine on rapidly and slowly conducting axons of rat corpus callosum. Exp. Neurol. 79 (3), 808–820. 10.1016/0014-4886(83)90044-4 6825765

[B47] Prineas J. W. Kwon E. E. Cho E. S. Sharer L. R. Barnett M. H. Oleszak E. L. (2001). Immunopathology of secondary-progressive multiple sclerosis. Ann. Neurol. 50 (5), 646–657. 10.1002/ana.1255 11706971

[B48] Redford E. J. Kapoor R. Smith K. J. (1997). Nitric oxide donors reversibly block axonal conduction: demyelinated axons are especially susceptible. Brain 120 (12), 2149–2157. 10.1093/brain/120.12.2149 9448570

[B49] Sa M. J. (2012). Physiopathology of symptoms and signs in multiple sclerosis. Arq. Neuropsiquiatr. 70 (9), 733–740. 10.1590/s0004-282x2012000900016 22990733

[B50] Sachs H. H. Bercury K. K. Popescu D. C. Narayanan S. P. Macklin W. B. (2014). A new model of cuprizone-mediated demyelination/remyelination. ASN Neuro 6 (5), 1759091414551955. 10.1177/1759091414551955 25290063 PMC4187018

[B51] Sakurai M. Kanazawa I. (1999). Positive symptoms in multiple sclerosis: their treatment with sodium channel blockers, lidocaine and mexiletine. J. Neurol. Sci. 162 (2), 162–168. 10.1016/s0022-510x(98)00322-0 10202981

[B52] Salzer J. Svenningsson A. Sundstrom P. (2010). Neurofilament light as a prognostic marker in multiple sclerosis. Mult. Scler. 16 (3), 287–292. 10.1177/1352458509359725 20086018

[B53] Seggie J. Berry M. (1972). Ontogeny of interhemispheric evoked potentials in the rat: significance of myelination of the corpus callosum. Exp. Neurol. 35 (2), 215–232. 10.1016/0014-4886(72)90148-3 5030850

[B54] Smith K. J. Kapoor R. Hall S. M. Davies M. (2001). Electrically active axons degenerate when exposed to nitric oxide. Ann. Neurol. 49 (4), 470–476. 10.1002/ana.96 11310624

[B55] Smith M. E. Sommer M. A. (1992). Association between cell-mediated demyelination and astrocyte stimulation. Prog. Brain Res. 94, 411–422. 10.1016/s0079-6123(08)61768-9 1287726

[B56] Srinivasan R. Sailasuta N. Hurd R. Nelson S. Pelletier D. (2005). Evidence of elevated glutamate in multiple sclerosis using magnetic resonance spectroscopy at 3 T. Brain 128 (5), 1016–1025. 10.1093/brain/awh467 15758036

[B57] Steinman L. (2001). Multiple sclerosis: a two-stage disease. Nat. Immunol. 2 (9), 762–764. 10.1038/ni0901-762 11526378

[B58] Stojic A. Bojcevski J. Williams S. K. Diem R. Fairless R. (2018). Early nodal and paranodal disruption in autoimmune optic neuritis. J. Neuropathol. Exp. Neurol. 77 (5), 361–373. 10.1093/jnen/nly011 29444299

[B59] Sturrock R. R. (1980). Myelination of the mouse corpus callosum. Neuropathol. Appl. Neurobiol. 6 (6), 415–420. 10.1111/j.1365-2990.1980.tb00219.x 7453945

[B60] Stys P. K. Ransom B. R. Waxman S. G. (1991). Compound action potential of nerve recorded by suction electrode: a theoretical and experimental analysis. Brain Res. 546 (1), 18–32. 10.1016/0006-8993(91)91154-s 1855148

[B61] Su Y. Wang Y. Zhou Y. Zhu Z. Zhang Q. Zhang X. (2017). Macrophage migration inhibitory factor activates inflammatory responses of astrocytes through interaction with CD74 receptor. Oncotarget 8 (2), 2719–2730. 10.18632/oncotarget.13739 27926507 PMC5356836

[B62] t Hart B. A. Gran B. WeissertEAE R. (2011). EAE: imperfect but useful models of multiple sclerosis. Trends Mol. Med. 17 (3), 119–125. 10.1016/j.molmed.2010.11.006 21251877

[B63] Tekkok S. B. Faddis B. T. Goldberg M. P. (2005). AMPA/kainate receptors mediate axonal morphological disruption in hypoxic white matter. Neurosci. Lett. 382 (3), 275–279. 10.1016/j.neulet.2005.03.054 15925103

[B64] Tekkok S. B. Goldberg M. P. (2001). Ampa/kainate receptor activation mediates hypoxic oligodendrocyte death and axonal injury in cerebral white matter. J. Neurosci. 21 (12), 4237–4248. 10.1523/JNEUROSCI.21-12-04237.2001 11404409 PMC6762765

[B65] Tekkok S. B. Ransom B. R. (2004). Anoxia effects on CNS function and survival: regional differences. Neurochem. Res. 29 (11), 2163–2169. 10.1007/s11064-004-6890-0 15662851

[B66] Tekkok S. B. Ye Z. Ransom B. R. (2007). Excitotoxic mechanisms of ischemic injury in myelinated white matter. J. Cereb. Blood Flow. Metab. 27 (9), 1540–1552. 10.1038/sj.jcbfm.9600455 17299453

[B67] Teunissen C. E. Khalil M. (2012). Neurofilaments as biomarkers in multiple sclerosis. Mult. Scler. 18 (5), 552–556. 10.1177/1352458512443092 22492131

[B68] Trapp B. D. Nave K. A. (2008). Multiple sclerosis: an immune or neurodegenerative disorder? Annu. Rev. Neurosci. 31, 247–269. 10.1146/annurev.neuro.30.051606.094313 18558855

[B69] Tseng C. H. Chen J. H. Muo C. H. Chang Y. J. Sung F. C. Hsu C. Y. (2015). Increased risk of ischaemic stroke amongst patients with chronic osteomyelitis: a population-based cohort study in Taiwan. Eur. J. Neurol. 22 (4), 633–639. 10.1111/ene.12387 24602152

[B70] Vandenbark A. A. Meza-Romero R. Benedek G. Offner H. (2019). A novel neurotherapeutic for multiple sclerosis, ischemic injury, methamphetamine addiction, and traumatic brain injury. J. Neuroinflammation 16 (1), 14. 10.1186/s12974-018-1393-0 30683115 PMC6346590

[B71] Vandenbark A. A. Meza-Romero R. Wiedrick J. Gerstner G. Headrick A. Kent G. (2021). Brief report: enhanced drα1-mMOG-35-55 treatment of severe EAE in MIF-1-deficient male mice. Cell Immunol. 370, 104439. 10.1016/j.cellimm.2021.104439 34607646 PMC8920317

[B72] Vandenbark A. A. Meza-Romero R. Wiedrick J. Gerstner G. Seifert H. Kent G. (2022). Near Cure" treatment of severe acute EAE in MIF-1-deficient female and male mice with a bifunctional MHCII-derived molecular construct. Cell Immunol. 378, 104561. 10.1016/j.cellimm.2022.104561 35738135 PMC9714992

[B73] Vandenbark A. A. Rich C. Mooney J. Zamora A. Wang C. Huan J. (2003). Recombinant TCR ligand induces tolerance to myelin oligodendrocyte glycoprotein 35-55 peptide and reverses clinical and histological signs of chronic experimental autoimmune encephalomyelitis in HLA-DR2 transgenic mice. J. Immunol. 171 (1), 127–133. 10.4049/jimmunol.171.1.127 12816990

[B74] Vercellino M. Merola A. Piacentino C. Votta B. Capello E. Mancardi G. L. (2007). Altered glutamate reuptake in relapsing-remitting and secondary progressive multiple sclerosis cortex: correlation with microglia infiltration, demyelination, and neuronal and synaptic damage. J. Neuropathol. Exp. Neurol. 66 (8), 732–739. 10.1097/nen.0b013e31812571b0 17882017

[B75] Wallin M. T. Culpepper W. J. Campbell J. D. Nelson L. M. Langer-Gould A. Marrie R. A. (2019). The prevalence of MS in the United States: a population-based estimate using health claims data. Neurology 92 (10), e1029–e1040. 10.1212/WNL.0000000000007035 30770430 PMC6442006

[B76] Wiedrick J. Meza-Romero R. Gerstner G. Seifert H. Chaudhary P. Headrick A. (2021). Sex differences in EAE reveal common and distinct cellular and molecular components. Cell Immunol. 359, 104242. 10.1016/j.cellimm.2020.104242 33190849 PMC7770093

[B77] Witte M. E. Mahad D. J. Lassmann H. van Horssen J. (2014). Mitochondrial dysfunction contributes to neurodegeneration in multiple sclerosis. Trends Mol. Med. 20 (3), 179–187. 10.1016/j.molmed.2013.11.007 24369898

[B78] Yadav V. Bourdette D. N. Bowen J. D. Lynch S. G. Mattson D. Preiningerova J. (2012). Recombinant T-cell receptor ligand (RTL) for treatment of multiple sclerosis: a double-blind, placebo-controlled, Phase 1, dose-escalation study. Autoimmune Dis. 2012, 954739. 10.1155/2012/954739 22548151 PMC3328144

[B79] Yang L. Liu Z. Ren H. Zhang L. Gao S. Ren L. (2017). DRα1-MOG-35-55 treatment reduces lesion volumes and improves neurological deficits after traumatic brain injury. Metab. Brain Dis. 32 (5), 1395–1402. 10.1007/s11011-017-9991-6 28303450 PMC5600636

[B80] Yuan A. Rao M. V. Veeranna Nixon R. A. (2012). Neurofilaments at a glance. J. Cell Sci. 125 (14), 3257–3263. 10.1242/jcs.104729 22956720 PMC3516374

